# Cross-border differences in the prevalence and risk factors for carriage of antimicrobial resistance in children attending daycare centers: a point prevalence study in the Netherlands and Belgium

**DOI:** 10.1186/s12879-024-08996-9

**Published:** 2024-01-24

**Authors:** Sara Dequeker, Mitch van Hensbergen, Casper D. J. den Heijer, Wouter Dhaeze, Stijn F. H. Raven, Helen Ewalts-Hakkoer, Paulien Tolsma, Ina Willemsen, Karine J. van Drunen-Kamp, Krista van der Slikke-Verstraten, Herman Goossens, Marjolein F. Q. Kluytmans-van den Bergh, Christian J. P. A. Hoebe, on behalf of the i-4-1-Health Study Group, Lieke van Alphen, Nicole van den Braak, Caroline Broucke, Anton Buiting, Liselotte Coorevits, Jeroen Dewulf, Bram Diederen, Inge Gyssens, Casper Jamin, Patricia Jansingh, Jan Kluytmans, Stefanie van Koeveringe, Sien De Koster, Christine Lammens, Isabel Leroux-Roels, Hanna Masson, Ellen Nieuwkoop, Anita van Oosten, Natascha Perales Selva, Merel Postma, Veroniek Saegeman, Paul Savelkoul, Annette Schuermans, Nathalie Sleeckx, Tijs Tobias, Jacobien Veenemans, Dewi van der Vegt, Martine Verelst, Carlo Verhulst, Pascal De Waegemaeker, Veronica Weterings, Clementine Wijkmans, Patricia Willemse Smits

**Affiliations:** 1 Department of Epidemiology and public health, Sciensano, Brussels, Belgium; 2 Agency for Care and Health, Infection Prevention and Control, Government of Flanders, Brussels, Belgium; 3 Department of Sexual Health, Infectious Diseases and Environmental Health, Living Lab Public Health, South Limburg Public Health Service, Heerlen, The Netherlands; 4 Department of Social Medicine, Care and Public Health Research Institute (CAPHRI), Maastricht University, PO Box 616, 6200 MD Maastricht, The Netherlands; 5 Department of Infectious Diseases, Public Health Service region Utrecht, Zeist, The Netherlands; 6 Public Health Service Hart voor Brabant, Tilburg, The Netherlands; 7 Public Health Service Brabant-Zuidoost, Eindhoven, The Netherlands; 8 Contrain Infectiepreventiecoach, Breda, The Netherlands; 9 Amphia Hospital, Breda, The Netherlands; 10 Publich Health Service Zeeland, Goes, The Netherlands; 11 University of Antwerp, Antwerp, Belgium; 12 UMC Utrecht, Utrecht, the Netherlands; 13 Department of Medical Microbiology, Infectious Diseases and Infection Prevention, Care and Public Health Research Institute (CAPHRI), Maastricht University Medical Centre (MUMC+), PO Box 5800, 6202 AZ Maastricht, The Netherlands

**Keywords:** ESBL-E, CipR-E, Children, Day care centres, Belgium, The Netherlands

## Abstract

**Background:**

Day care centres (DCCs) are ideal settings for drug-resistant bacteria to emerge. Prevalence numbers of faecal carriage of antimicrobial resistant bacteria in these settings are rare. We aimed to determine the prevalence of faecal antimicrobial resistant bacteria carriage in children attending DCCs and to assess and identify infection risk factors within DCCs in The Netherlands and Belgium.

**Methods:**

A point-prevalence study was conducted in 28 Dutch (499 children) and 18 Belgian (448 children) DCCs. Stool samples were taken from the children’s diapers and a questionnaire was filled in by their parents. Hygiene related to stool and toilet use, hygiene related to food, environmental contamination, hand hygiene and hygiene guidelines were assessed conform a standardized questionnaire by the infection prevention and control expert visiting the DCC. Multilevel logistical regression analyses were used to define which characteristics predicted the presence of extended-spectrum beta-lactamase-producing *Enterobacterales* (ESBL-E), carbapenemase-producing *Enterobacterales* (CPE), vancomycin-resistant enterococci (VRE), and ciprofloxacin-resistant *Enterobacterales* (CipR-E).

**Results:**

The ESBL-E prevalence was 16% (*n* = 71) in Belgium and 6% (*n* = 30) in the Netherlands. The CipR-E prevalence was 17% (*n* = 78) in Belgium and 8% (*n* = 38) in the Netherlands. Antimicrobial use (RR: 0.30; 95% CI: 0.33–0.48) and hospital admissions (RR: 0.37; 95% CI: 0.25–0.54) were lower in the Netherlands. Children travelling to Asia were at higher risk of being an ESBL-E carrier. Children using antimicrobials were at higher risk of being a CipR-E carrier. Cleaning the changing mat after each use was found as a protective factor for CipR-E carriage.

**Conclusions:**

We established a significant difference in ESBL-E and CipR-E carriage and antimicrobial use and hospital admissions between the Netherlands and Belgium among children attending DCCs. The differences between both countries should be further studied to improve the policy on anti-microbial use and hospital admissions in children.

**Supplementary Information:**

The online version contains supplementary material available at 10.1186/s12879-024-08996-9.

## Introduction

Infants (< 18 months old) have more and closer interpersonal contacts than older children and adults. Additionally, they are not yet aware of personal hygiene, resulting in a higher chance of faecal-oral transmission of microorganisms within this age group [1]. For this reason, bacteria can pass easily among children in day care centres (DCCs) [2] and, subsequently, children attending DCCs are at higher risk for infections [1]. Moreover, the declining maternal immunity, in combination with an immature immune system, makes infants even more susceptible to infections and, therefore, more likely to receive antimicrobial treatment. This treatment exerts selection pressure on the intestinal microbiota and promotes carriage of resistant bacteria [1]. This makes DCCs ideal settings for drug-resistant strains of bacteria to emerge [2]. Still, few studies have focussed on the prevalence and risk factors for resistant bacteria in young children. An overall prevalence of extended-spectrum beta-lactamase (ESBL)-producing bacteria in DCC-attending children of 4.0–16.8% was established in the Netherlands, in France and in Sweden between 2012 and 2016 [1, 3–5]. In Sweden, a more than six-fold increase in the prevalence of ESBL-producing *Enterobacterales* (ESBL-E) was determined in 2016 compared with a similar study conducted 6 years earlier [5].

Finally, the infectious disease burden not only concerns the attending child. Infectious pathogens, including their antimicrobial resistance properties, may transmit via children, caretakers, parents and families [3, 6] into the society at large. Therefore, it is important to intensify our efforts in infection control and antimicrobial stewardship [7] and to support DCCs in their efforts to control and prevent infectious disease transmission within their facility.

In this point prevalence study we measured different infection risk factors in a standardized way based on the Infection RIsk Scan (IRIS), a tool to measure the quality of infection control and antimicrobial use, both at individual and DCC group level [8]. This study included DCCs in three southern provinces of the Netherlands (Limburg, Noord-Brabant and Zeeland) and the five Flemish provinces of Belgium (Antwerpen, Limburg, Oost-Vlaanderen, Vlaams-Brabant and West-Vlaanderen). This study was part of a larger Interreg project, which aimed at broadening the knowledge regarding antimicrobial resistance and use in different healthcare and veterinary settings among cross-border countries, specifically Belgium and the Netherlands [9].

The study aimed to determine the prevalence of faecal antimicrobial resistant bacteria carriage in children attending DCCs and to assess and identify infection risk factors within DCCs in the Netherlands and Belgium.

## Methods

### Design

A cross-sectional point prevalence study was performed between October 2018 and February 2019, as part of the Dutch cross border i-4-1-Health study [10]. The results of the study are reported according to the STrengthening the Reporting of OBservational studies in Epidemiology (STROBE) statement [11].

### Sampling method

The sampling method differed between both countries.

In the five Flemish provinces of Belgium, there were ~ 2.300 DCCs at the time of recruitment, of which ~ 300 had room for at least 40 children. The aim was to include 20 DCCs with room for at least 40 children in the study. Therefore, four DCCs per province were selected at random. If a DCC refused to participate, the DCC following the non-participating DCC in the list, was invited to participate.

In the three southern provinces of the Netherlands, there were ~ 1.900 DCCs at the time of recruitment. A convenience sampling method was used to recruit 3–4 DCC per public health service (GGD).

### Participants

DCCs from the Dutch-Belgian cross-border region were invited to participate, of which 28 Dutch and 18 Belgian DCCs were included in the study. A total of 82 Dutch and 104 Belgian groups within these DCCs participated, including 59 infant groups (< 18 months old), 59 toddler groups (≥18 months and < 4 years old) and 67 vertical groups (both infants and toddlers).

Parents of all children attending these DCCs were asked to give informed consent to include their child in the study. Continent children were excluded. Another exclusion criterion was a long transport (> 72 hours) of the sample to the laboratory (*n* = 3).

## Data collection

### Faecal samples and microbiological analyses

To determine the (point) prevalence of antimicrobial resistance, stool samples were taken from the children’s diapers (FecalSwab® with Cary-Blair transportmedium, Copan, Brescia, Italia). Each child could only provide one stool sample. Dutch samples were sent to the Microvida Laboratory for Medial Microbiology Amphia Hospital (Breda, the Netherlands) and Belgian samples to the microbiological laboratory of the University Hospital Antwerp (Antwerp, Belgium). For the microbiological analyses a standardized protocol was used in both countries [10]. Faecal samples were selectively cultured (ChromID® ESBL/CARBA/OXA-48/VRE, bioMérieux; in-house McC ciprofloxacin) after non-selective pre-enrichment (TSB, Copan Italy, Bresica, Italy) to identify vancomycin-resistant enterococci (VRE), ESBL-E, ciprofloxacin-resistant *Enterobacterales* (CipR-E) and carbapenemase-producing *Enterobacterales* (CPE).

### Child related risk factors

Parents completed a short questionnaire to measure child related risk factors for carriage of antimicrobial resistance (AMR). Included risk factors, selected based on expert consensus within our study group, were: age, days per week attending the DCC, gender, antimicrobial use, hospital admission, parental occupation, animal contact and travel abroad (Table 1).

**Table 1:** Child characteristics, median and IQR or frequency per country and odds ratio’s or risk ratio's

Characteristic	Belgium *N* = 448		The Netherlands *N* = 499		Belgium reference category	
	Median	(IQR)	Median	(IQR)	Odds ratio	95% CI (wald)
Age (years)^a,f^	1	(1–2)	2	(1–2)	**1.35**	**1.07–1.71**
Days per week attending the DCC^a,f^	4	(3–5)	2	(1–3)	**0.03**	**0.02–0.04**
	**n**	**(%)**	**n**	**(%)**	**Risk ratio**	**95% CI (wald)**
Gender (female)^a,f^	232	(52.5)	243	(48.8)	1.08	0.95–1.23
Antimicrobial use (< 6 months)^a,f^	232	(52.5)	104	(20.9)	**0.40**	**0.33–0.48**
Hospital admission (< 6 months)^b,g^	80	(18.2)	33	(6.6)	**0.37**	**0.25–0.54**
Parental occupation						
At least one parent is working in health care^c,h^	124	(27.7)	138	(27.7)	1.0	0.81–1.23
Animal contact (< 6 months)^d,f^	322	(73.4)	423	(84.9)	**1.16**	**1.08–1.24**
Pet^e,f^	300	(68.5)	368	(73.7)	1.08	0.99–1.17
Zoo^e,f^	113	(25.8)	213	(42.8)	**1.66**	**1.37–2.00**
Livestock^e,f^	24	(5.5)	58	(11.7)	**2.13**	**1.34–3.36**
Travel abroad (< 6 months)^b,f^	242	(55.0)	277	(55.6)	1.01	0.90–1.13
Africa^b,f^	9	(2.1)	8	(1.6)	0.79	0.31–2.02
Asia^b,f^	6	(1.4)	4	(0.8)	0.59	0.17–2.07
Europe^b,f^	229	(52.1)	261	(52.4)	1.00	0.89–1.14
Latin-America^b,f^	0	(0.0)	10	(2.0)	**0.98**	**0.97–0.99**
North-America^b,f^	6	(1.4)	2	(0.4)	0.29	0.06–1.45

Belgium: *N* = 448. ^a^*n* = 442, ^b^*n* = 440, ^c^*n* = 448, ^d^*n* = 439, ^e^*n* = 438

The Netherlands: *N* = 499,^f^*n* = 498, ^g^*n* = 497, ^h^*n* = 499

*DCC* day care centre, *95% CI* 95% confidence interval

### DCC infection risk factors (in general)

Hygiene related to stool and toilet use, hygiene related to food, environmental contamination, hand hygiene and hygiene guidelines were assessed by the infection prevention and control (IPC) expert visiting the DCC in a standardized way based on the IRIS (Table 2) [8].

**Table 2:** Group characteristics, frequency per country and risk ratio’s

Indicators	Belgium *N* = 104		The Netherlands *N* = 81		Belgium reference category	
	*n*	(%)	*n*	(%)	Risk ratio	95% CI (wald)
Toilet hygiene						
*Toilet at child height (or toilet seat reducer with step)*^a,f, **^	63	(96.9)	53	(96.4)	0.99	0.93–1.06
*Sink at child height*^b,f,**^	38	(54.3)	55	(100)	**1.84**	**1.49–2.28**
*Liquid soap available at this sink*^c,f, **^	9	(13.0)	46	(83.6)	**6.41**	**3.45–11.92**
The changing mat cover is intact and easy to clean	103	(99.0)	76	(93.8)	0.95	0.89–1.01
Changing mat is cleaned after each use (or a protective pad is used)^d,g^	54	(59.3)	64	(81.0)	**1.37**	**1.12–1.67**
Used diapers are immediately put in the diaper container^e,g^	100	(99.0)	71	(89.9)	**0.91**	**0.84–0.98**
Liquid soap available at the sink next to the changing mat^h^	96	(92.3)	79	(98.8)	**1.08**	**1.02–1.15**
Liquid soap available at the sink at the staff toilet^h^	94	(90.4)	78	(97.5)	1.07	1.00–1.14
*No potties are used*^b,f,**^	16	(22.9)	35	(63.6)	**2.78**	**1.73–4.47**
Disposable paper towels are available at all sinks	85	(81.7)	53	(65.4)	0.80	0.67–0.96
Food hygiene						
Maximal temperature of the fridge > = 4 °C (Belgium) or > = 7 °C (the Netherlands)^I,g^	23	(29.9)	44	(55.7)	**1.86**	**1.26–2.77**
*Child-dedicated bottles and teats*^j,p,*^	56	(93.3)	58	(100.0)	1.07	1.00–1.15
*After use, the bottles are immediately rinsed with cold water and dried upside down*^k,p,*^	14	(25.0)	44	(75.9)	**3.03**	**1.88–4.89**
*Only formula in powdered form are accepted*^l,p,*^	59	(100.0)	57	(98.3)	0.98	0.95–1.02
Food preparation is separated from the changing area	100	(96.2)	81	(100)	1.04	1.00–1.08
*Breast milk is defrosted in the fridge*^*m,q,$*^	40	(78.4)	53	(98.2)	**1.25**	**1.08–1.45**
No foods have passed their expiry date^n^	83	(86.5)	69	(85.2)	0.99	0.87–1.11
Cleaning schedule for the kitchen^d^	57	(62.6)	72	(88.9)	**1.42**	**1.19–1.69**
Dish cloths, towels and tea towels are not visually soiled^o^	89	(96.7)	72	(88.9)	0.92	0.84–1.00
Hand hygiene and preconditions						
*Hand hygiene education for children is given*^r,q,**^	22	(34.4)	36	(66.7)	**1.94**	**1.32–2.86**
*Children wash their hands after going to the toilet*^r,w,**^	14	(21.9)	39	(81.3)	**3.71**	**2.29–6.02**
*Children wash their hands after playing outside*^s,x,**^	32	(45.1)	44	(88.0)	**1.95**	**1.48–2.57**
*Children wash their hands before eating*^s,f,**^	20	(28.2)	44	(80.0)	**2.84**	**1.91–4.21**
Staff wash their hands after changing a diaper or cleaning the nose/bum of a child^t,g^	25	(26.6)	60	(76.0)	**2.86**	**2.00–4.09**
Staff wash their hands after going to the toilet^u,y^	13	(59.1)	68	(100.0)	**1.69**	**1.20–2.40**
Staff does not wear rings^v,h^	59	(57.8)	16	(20.0)	**0.35**	**0.22–0.55**
Staff does not wear wrist jewellery^v,h^	59	(57.8)	22	(27.5)	**0.48**	**0.32–0.70**

Belgium: *N* = 104. ^a^*n* = 65, ^b^*n* = 70, ^c^*n* = 69, ^d^*n* = 91, ^e^*n* = 101, ^i^*n* = 77; ^j^*n* = 60, ^k^*n* = 56, ^l^*n* = 59, ^m^*n* = 51, ^n^*n* = 96, ^o^*n* = 92, ^r^*n* = 64, ^s^*n* = 71, ^t^*n* = 94, ^u^*n* = 22, ^v^*n* = 102

The Netherlands: *N* = 81. ^f^*n* = 55, ^g^*n* = 79,^h^*n* = 80, ^p^*n* = 58, ^q^*n* = 54, ^w^*n* = 48, ^x^*n* = 50, ^y^*n* = 68

* only observed in groups where infants (< 18 months old) are present, and therefore excluded from the multilevel model

** only observed in groups where toddlers (≥18 months and < 4 years old) are present, and therefore excluded from the multilevel model

*DCC* day care centre, 95% *CI* 95% confidence interval

**Fig. 1:**
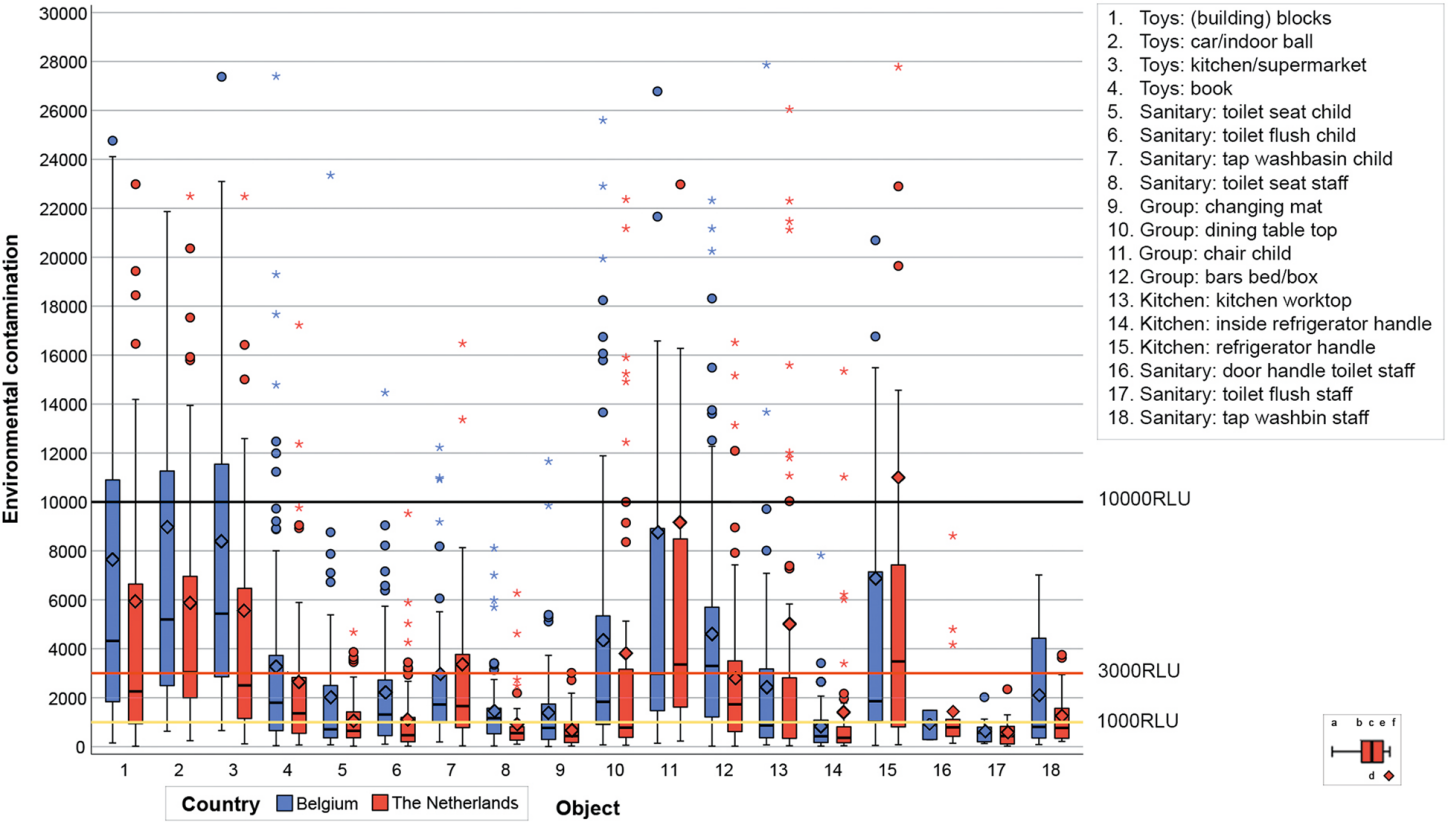
Environmental contamination, mean, median and IQR per measured object, per country. Legend boxplot: a. maximum (without outliers, 1.5x interquartile range), b. 75th percentile (P75), c. median, d. mean, e. 25th percentile (P25), f. minimum (without outliers, 1.5x interquartile range)

Adenosine triphosphate (ATP) assessment was used to measure environmental contamination. ATP bioluminescence detects the bioburden present, which is a quick way to assess the cleanliness of an environmental surface. This assessment provides a quick and sensitive test that can detect whether cleaning is (in) adequate [12]. The Clean-Trace Luminometer (3 M) was used for the ATP assessments and its output was expressed in relative light units (RLU). ATP assessments were conducted on 15 pre-defined surfaces by the IPC expert visiting the DCC (Fig. 1). These pre-defined surfaces were categorized into four groups: (1) Toys, (2) Sanitary, (3) General materials in the group and (4) Kitchen. These surfaces were discussed in an IPC expert meeting and chosen because they were frequently touched by staff or children, or should by definition always be clean. The level of environmental contamination was categorized into clean (< 1000 RLU), intermediate (≥1000 and < 3000 RLU), dirty (≥3000 and < 10,000 RLU), and extremely dirty (≥10,000 RLU); which were scored as 0, 1, 2 and 3 respectively [8, 13]. Because not all pre-defined surfaces were (always) available at the moment of sampling, an average score per category was calculated and rounded to the closest integer, presenting an overall level of environmental contamination per category, as long as at least one surface was sampled within the category.

## Data analysis

Descriptive analyses were used to show the presence of infection risk factors in DCCs and AMR risk factors in children. A prevalence of children in DCCs carrying ESBL-E, CPE, VRE, and CipR-E was calculated, by dividing the number of children testing positive (based on phenotype) by the total number of included children. Risk ratios (RR) (binomial variables) or univariate logistical regression (odds ratio: OR) (continuous variables) were used to assess the difference between the measured variables between both countries.

Multilevel logistical regression analyses were used to define which child/DCC characteristics predicted the presence/absence of ESBL-E, CPE, VRE, and CipR-E. Multilevel analyses take the hierarchical structure of the data into account [14]. A two-level model was used: (1) child level (Level 1) and (2) DCC group level (Level 2). This means that children are nested within groups. Variables at child level were age, days per week attending the DCC, gender, antimicrobial use, hospital admission, parental occupation, animal contact and travel abroad. Variables at group level were the country and variables related to toilet hygiene, food hygiene, hand hygiene and environmental contamination [15]. For the individual data (questionnaires) the missingness was considered as completely at random (MCAR), as incomplete questionnaires were totally at random. For the data on group level, the missingness was considered at random (MAR), as the missing data was mostly linked to the group itself. No imputation techniques were performed, but a complete case analysis was used.

Two multilevel models were built, one with ESBL-E as the dependent variable and one with CipR-E as the dependent variable. For both models, predictors were first assessed in univariate models (see Additional File 1) with the relevant dependent variable and checked for multicollinearity. Independent variables significant at the 0.25 level were included in the final model. The variable “Staff wash their hands after going to the toilet” was excluded from the model because of the high correlation with “Liquid soap available at the sink at the staff toilet”. The general variables “animal contact” and “travel abroad” were disaggregated into “place where the animal lives” and “travelling to continent”. Some variables were only observed on infant groups (< 18 months old) (e.g. only formulae in powdered form are accepted), while other variables were only observed on toddler groups (≥18 months and < 4 years old) (e.g. no potties are used). Because these variables introduced a lot of missing values and reduced the number of observations in the model, only the variables who were available for all kind of group types were included in the models.

First, a null (intercept only) model with no predictors at any level was built. The null model serves as comparison for other models and provides information on how much variation in the outcome exists between level-2 units [14]. Second, child characteristics (Level 1 variables) were added to the model. Finally, a third model was built, including risk factors at DCC group level (Level 2 variables). The likelihood ratio (χ^2^) test was used to test the improvement of fit for each model [14].

All analyses were performed in SAS Enterprise Guide 7.1 (SAS Institute Inc., Cary, North Carolina, USA). A *p*-value of 0.05 was considered statistically significant.

## Results

### Participant characteristics

A total of 449 Dutch and 448 Belgian children were included in the study. Antimicrobial use in the previous 6 months was significantly lower among Dutch children (21%, *n* = 104) compared to Belgian children (53%, *n* = 232) (RR: 0.30; 95% CI: 0.33–0.48). Hospital admission in the previous 6 months was significantly lower in Dutch children (7%, *n* = 33) compared to Belgian children (18%, *n* = 80) (RR: 0.37; 95% CI: 0.25–0.54) (see Table 2). Other child characteristics per country can be found in Table 1.

### DCC group characteristics

The DCC group characteristics are presented in Table 2. Some significant differences could be observed between both countries. In toddler DCC groups, more attention and education about hand hygiene for children was given in the Netherlands, compared to Belgium. In the Netherlands, a sink at child height was present in more DCC groups for toddlers.

The compliance for hand hygiene among staff was significantly higher and significantly more attention was given to food hygiene in the Netherlands than in Belgium.

### Environmental contamination

In Fig. 1 a boxplot per country for each measured object was made, expressed in RLU. Differences between both countries were observed. The environmental contamination was higher for the toys and the general materials in the groups than for the other surfaces. For most surfaces the median between both countries was comparable, except for the toys (OR: 0.30; 95%CI: 0.16–0.53) and general materials in the groups (OR: 0.39; 95%CI: 0.22–0.71) who seemed to be less contaminated in Dutch DCCs than in Belgian DCCs.

### Antimicrobial resistance

In the Netherlands, 6% (*n* = 30, range per DCC: 0–25.6%) of the children were carriers of ESBL-E, compared to 16% (*n* = 71, range per DCC: 0–50%) of the children in Belgium, which was lower (RR:0.38; 95%CI: 0.25–0.57). Eight percent (*n* = 38, range per DCC: 0–33.3%) of the Dutch children were carriers of CipR-E, compared to 17% (*n* = 78, range per DCC: 0–50%) of the Belgian children, which was significantly lower (RR: 0.44; 95%CI: 0.30–0.63). No carriers of CPE or VRE were found.

### Multilevel model – ESBL-E

The first model, the null model, showed a log odds of being a carrier of ESBL-E of − 2.64 (SE = 0.21), which is the odd to be a carrier of ESBL-E across all groups (*n* = 186). The intraclass correlation coefficient (ICC) revealed that 34% of the variance to be a carrier of ESBL-E was explained by the groups within the DCC. The second model included children’s demographics and characteristics. This model indicated that, on average, children who travelled to Asia within the previous 6 months (log odds: 3.88 – SE: 1.28) and children who received an antimicrobial treatment within the previous 6 months (log odds: 0.65 – SE: 0.33) had a statistically significant increased risk to be an ESBL-E carrier. Other covariates were not associated with carriage of ESBL-E. The ward level variance of carriage increased from 34 to 45% after adjusting for children’s demographics and characteristics.

The third model included variables at DCC group level. This model indicated that on average, only children who travelled to Asia in the previous 6 months (log odds: 3.07– SE: 1.19) had a higher odds to be a carrier of ESBL-E. The ward level variance was reduced from 45 to 28% after adjusting for the level 2 variables. Complete results for all models are available in Table 3.

**Table 3:** Risk factors for carriage of extended-spectrum beta-lactamase-producing *Enterobacterales*: multilevel models

Extended-spectrum beta-lactamase-producing *Enterobacterales*	Model 0	Model 1		Model 2	
N used	947	744		545	
	Intercept only	With level 1 characteristics (detailed travel/animal)		With level 2 characteristics (detailed travel/animal)	
		Estimate (Standard error)	Odds Ratio (95% CI)	Estimate (Standard error)	Odds Ratio (95% CI)
Intercept	−2.64** (0.21)	−3.54** (0.80)		−16.98 (637.05)	
Days per week attending the DCC		0.19 (0.15)	1.21 (0.91–1.61)	0.16 (0.20)	1.17 (0.78–1.75)
Antimicrobial use (< 6 months)		0.65* (0.33)	1.92 (0.99–3.69)	0.13 (0.41)	1.14 (0.51–2.55)
Animal contact (< 6 months) – at home		−0.65 (0.53)	0.52 (0.19–1.48)	−0.04 (0.64)	0.96 (0.27–3.40)
Animal contact (< 6 months) – zoo		−0.11 (0.36)	0.90 (0.45–1.82)	0.10 (0.43)	1.11 (0.48–2.59)
Animal contact (< 6 months) – live stock		−0.90 (0.72)	0.41 (0.10–1.65)	−1.78 (1.16)	0.17 (0.02–1.66)
Travel abroad (< 6 months) – Africa		0.54 (1.06)	1.72 (0.21–13.88)	0.60 (1.11)	1.82 (0.21–15.99)
Travel abroad (< 6 months) – Asia		3.88* (1.28)	**48.65 (3.93–602.89)**	3.07* (1.19)	**21.56 (2.04–227.58)**
Travel abroad (< 6 months) – Europe		0.19 (0.32)	1.21 (0.64–2.29)	0.29 (0.38)	1.34 (0.64–2.80)
Country				0.49 (0.74)	1.64 (0.38–7.06)
ATP sanitary				0.02 (0.44)	1.02 (0.43–2.42)
ATP group				−0.23 (0.39)	0.79 (0.37–1.70)
Changing mat is cleaned after each use				−0.43 (0.58)	0.65 (0.21–2.02)
Used diapers are immediately put in the diaper container				13.67 (637.05)	> 999.9 (/ - /)
Liquid soap available at the sink at the staff toilet				−1.34 (0.85)	0.26 (0.05–1.39)
Maximum temperature of fridge > = the national guideline				−0.66 (0.54)	0.52 (0.18–1.50)
Cleaning schedule for the kitchen				0.68 (0.61)	1.97 (0.60–6.51)
Dish cloths, towels and tea towels are not visually soiled				1.47 (0.96)	4.34 (0.66–28.61)
Staff wash their hands after changing a diaper of cleaning the nose/bum of a child				−0.01 (0.58)	0.99 (0.32–3.07)
Staff does not wear rings				−0.23 (0.52)	0.80 (0.29–2.21)
*Error variance*					
Level − 2 Intercept	1.68* (0.55)	2.74* (1.19)		1.28 (0.87)	
*Model Fit*					
-2 Log-likelihood	608.91	389.57		261.16	
∆ log likelihood (∆df)		219.34 (9)		128.41 (11)	
*p*-value		< 0.001		< 0.001	
ICC_DCCgroups_	0.34	0.45		0.28	
AIC	612.91	409.57		303.16	

*AIC* Aikake Information Criterion, *ATP* Adenosine triphosphate, *DCC* day care centre, *ICC* interclass correlation coefficient, *95% CI* 95% confidence interval

*p < 0.05, **p < 0.001

### Multilevel model – CipR-E

The first model, the null model, showed a log odds of being a carrier of CipR-E of − 2.21 (SE = 0.16), which is the odd to be a carrier of CipR-E across all groups (*n* = 186). The ICC revealed that 21% of the variance to be a carrier of CipR-E bacteria was explained by the groups within the DCC. The second model included children’s demographics and characteristics. This model indicated that, on average, children who travelled to Asia within the previous 6 months (log odds: 1.53 – SE: 0.74) had a higher odds to be a carrier of CipR-E. Other covariates were not significantly associated with carriage of CipR-E. The ward level variance of carriage was reduced from 21 to 13% after adjusting for children’s demographics and characteristics.

The third included variables at DCC group level. This model indicated that children who received an antimicrobial treatment within the previous 6 months (log odds: 0.53 – SE: 0.26) significantly had a higher odds to be carrier of CipR-E. Children attending DCC groups where the changing mat was cleaned after each use (log odds: -0.75 – SE: 0.34) significantly had less chance to be a carrier of CipR-E. The ward level variance was further reduced to 9% after adjusting for the level 2 variables. Complete results for all models are available in Table 4.

**Table 4:** Risk factors for carriage of ciprofloxacin-resistant *Enterobacterales*: multilevel models

Ciprofloxacin-resistant *Enterobacterales*	Model 0	Model 1b		Model 2b	
N used	947	935		716	
	Intercept only	With level 1 characteristics		With level 2 characteristics	
		Estimate (Standard Error)	Odds Ratio (95% CI)	Estimate (Standard Error)	Odds Ratio (95% CI)
Intercept	−2.21** (0.16)	− 2.52** (0.38)		−3.16* (1.20)	
Days per week attending the DCC		0.14 (0.09)	1.15 (0.96–1.38)	−0.11 (0.14)	0.89 (0.68–1.18)
Antimicrobial use (< 6 months)		0.29 (0.22)	1.34 (0.86–2.09)	0.53* (0.26)	**1.70 (1.01–2.84)**
Animal contact (< 6 months) – at home		−0.23 (0.23)	0.79 (0.51–1.25)	−0.06 (0.27)	0.94 (0.55–1.60)
Animal contact (< 6 months) – zoo		− 0.13 (0.24)	0.88 (0.55–1.41)	0.13 (0.27)	1.14 (0.67–1.94)
Travel abroad (< 6 months) – Asia		1.53* (0.74)	**4.63 (1.08–19.90)**	0.24 (1.26)	1.27 (0.11–14.98)
Country				0.95 (0.49)	2.57 (0.98–6.74)
Changing mat is intact and easy to clean				0.65 (0.82)	1.92 (0.38–9.65)
Changing mat is cleaned after each use				−0.75* (0.34)	**0.48 (0.25–0.92)**
Maximum temperature of fridge > = the national guideline				−0.04 (0.31)	0.96 (0.53–1.76)
Food within expire date				−0.48 (0.40)	0.62 (0.28–1.37)
Cleaning schedule for the kitchen				0.28 (0.34)	1.32 (0.67–2.59)
Dish cloths, towels and tea towels are not visually soiled				0.57 (0.54)	1.77 (0.61–5.16)
Staff wash their hands after changing a diaper of cleaning the nose/bum of a child				0.30 (0.32)	1.35 (0.72–2.54)
Staff does not wear rings				0.39 (0.33)	1.47 (0.77–2.81)
Staff does not wear wrist jewellery				0.27 (0.31)	1.31 (0.72–2.41)
*Error variance*					
Level − 2 Intercept	0.88* (0.38)	0.50 (0.32)		0.32 (0.31)	
*Model Fit*					
-2 Log-likelihood	692.76	671.27		458.89	
∆ log likelihood (∆df)	2	21.49 (5)		212.38 (10)	
*p*-value		0.001		< 0.001	
ICC_DCCgroups_	0.21	0.13		0.09	
AIC	696.76	685.27		523.14	

*AIC* Aikake Information Criterion, *ATP* Adenosine triphosphate, *DCC* day care centre, *ICC* interclass correlation coefficient, *95% CI* 95% confidence interval

*p < 0.05, **p < 0.001

## Discussion

### Main results

In children attending DCCs we showed an overall ESBL-E prevalence of 16% in Belgium versus 6% in the Netherlands, and an overall CipR-E prevalence of 17% in Belgium versus 8% in the Netherlands. The use of antimicrobial agents and hospital admissions among children attending DCCS was significantly higher in Belgium, compared to the Netherlands. For both child and DCC group characteristics, some significant differences could be observed between both countries. The final multilevel models indicated that, children who travelled to Asia within the previous 6 months had a higher odds to be a carrier of ESBL-E, whereas children who received an antimicrobial treatment within the previous 6 months, had a higher odds to be a CipR-E carrier. Attending DCC groups where the changing mat was cleaned after each use was found as a protective factor for carriership of CipR-E. No rectal carriage of VRE and of CPE was found in children attending a DCC on a phenotypical level.

- *Antimicrobial use and hospital admission*

Antimicrobial use differed significantly between the two countries. In 2014, 41% of the Belgian children (age < 16 years) received a systemic or ophthalmological antimicrobial, delivered by the home pharmacy (no hospital consumption) [16]. This included both antimicrobials prescribed in primary care and secondary care (paediatricians). A Dutch study showed that 15% of Dutch children (age ≤ 12 years), received at least one oral antimicrobial prescription per year during 2000–2010 in primary care [17]. The prescription rates for oral antimicrobials among Dutch children decreased significantly during the period 2006–2010 [17]. In both countries, the percentage of oral prescriptions was the highest in the youngest age groups (0-4 years) [16, 17]. Even though these studies do not describe the exact same population and more recent studies on antimicrobial use in young children in Belgium and the Netherlands are lacking, they seem to confirm that antimicrobial use among children differs between both countries. However, in this study parents were only questioned whether the child had received an antimicrobial agent in the previous 6 months, without asking further details about the drug used or when it was administered. Therefore, a recall bias might have been introduced. In addition, a period of 6 months may have been too long or the question too broad to show a significant effect.

There is also a significant difference in the number of hospital admissions in both countries. Additional information to explore further the difference in hospital admissions between both countries is missing. Only a general question was asked to the parents and no reason and/or date of admission were asked. Additionally, there was no distinction between ambulatory care and hospital admissions with at least one overnight stay. However, these forms of bias might have been introduced in both countries, allowing us to assume that there is indeed a substantial difference between the two countries.

Additionally, there are differences in the organization of DCCs for infants and toddlers, where Belgian DCCs have lower levels of preventive hygiene and have younger children who, on average, spend more days per week in day care. This suggests that cultural differences between both countries might play an important role in the use of antimicrobials and the emergence of antimicrobial resistance. We, therefore, recommend exploring further the difference in antimicrobial use and hospital admissions in children between the two countries more in-depth, in order to improve the policy on antimicrobial use and hospital admissions in children.

b. *Carriage of resistant bacteria*

Rectal carriage of ESBL-E and CipR-E was significantly higher in Belgium than in the Netherlands. In the larger Interreg project, similar results were found in different healthcare and veterinary settings [9, 18, 19]. In addition, the study done in the hospital setting showed that the number of ESBL-E carriers tested within 24 hours of hospital admission, representing a community carriage rate, was also higher in Belgium than in the Netherlands [9]. A possible hypothesis is that the results found in children attending DCCs and found in the other settings are a reflection of carrier status in the community. Unfortunately, a surveillance network is lacking in Belgium, which makes it difficult to monitor the epidemiology of carriage of antimicrobial resistance in the community [20]. Studies conducted between 2003 and 2018 reported higher ESBL carriage rates in the Belgian (11.6%) than in the Dutch community (5–10%) [21–25]. However, it should be noted that the prevalence in the Belgian community is based on one study with a sample of patients who were admitted to a geriatric unit in one teaching hospital in Belgium [24]. As the prevalence of faecal carriage among healthy individuals has increased (eight-fold) during the last two decades [26], the cited studies probably underestimate the current situation.

An alternative hypothesis that may explain the high prevalence in this study is the dissemination via direct contact as suggestions for household transmission have been described [3, 18]. Moreover, the results of molecular typing and whole genome sequencing observed transmission of ESBL-E in Belgian DCCs with a high ESBL-E prevalence [27].

Surprisingly, the carrier rate for CipR-E in children attending a DCC was so high in both countries. The use of ciprofloxacin and by extension fluoroquinolones in children under 16 years of age induces an increased risk of cartilage damage [28]. In practice, fluoroquinolones are only used in strict indication in children [16, 17, 29, 30]. This strengthens the hypothesis that the results established in children are a possible reflection of carrier status in the community.

Numerous enzymes associated with ESBL activity (mainly CTX-M) can diffuse easily due to their mobile genetic elements that mediate rapid dissemination. These are also linked with transfer of other genes that confer resistance to beta lactams as well as other antibacterial agents such as quinolones [26].

c. *Risk factors for carriage*

Risk factors for carriage have mainly been studied in adult hospitalized patients [3, 21]. Reported risk factors for carriage of ESBL-producing bacteria in healthy adults are travelling [22, 23], being owner of an animal [31], poor kitchen hygiene [23] and having a child attending a DCC [3]. Attending a DCC was determined to be a risk factor for carriage of ESBL-producing bacteria in children [3]. While looking at the children attending a DCC, being less than 1 year old and having paper towels available in the DCC increased the odds to be a carrier of ESBL. Prohibiting ill children from entering the DCC, extra supervision on handwashing of sick children and consistently reporting to local health authorities have been found as protective factors which lower the odds of being a carrier of ESBL [1]. Our study confirmed travelling to Asia as a risk factor for carriage of ESBL-producing bacteria in children attending a DCC. Despite recent travel to Asia being a clear significant risk factor for carriage of ESBL-E and CipR-E, this risk factor will not have a major impact on carrier status of resistant bacteria, as only a small number of children travel to Asia. To our knowledge, no studies evaluated risk factors for carriage of CipR-E in children.

It was unexpected to see that antimicrobial use did not emerge as a risk factor for ESBL-E and as a (borderline) significant risk factor for CipR-E. This might be explained by the lack of detailed information about the use of antimicrobials. A surveillance study in the community might give additional information about carriership of antimicrobial resistance in the community and children and provide insights in possible risk factors [25].

### Strengths and limitations

To the best of our knowledge, this is the first study that assessed ESBL-E, VRE, CPE and CipR-E carriage rates in children attending a DCC in Belgium and compared the results with the Netherlands. Additionally, this study has explored risk factors for AMR carriage, including ATP measurements. Besides the child related risk factors for AMR carriage, we included several pillars of infection prevention and control on the level of the DCC groups simultaneously, which can be seen as an added value. Two central laboratories analysed the samples, using a standardized protocol, which can be seen as another strength.

One of the limitations of the study is that, due to the lack of existing evidence, the included risk factors are mainly based on the experiences of the implementation of the IRIS in hospitals, national guidelines, and expert opinions. A lot of the risk factors on DCC group level, were measured through observation by an IPC expert. To improve the standardisation of data collection, all local IPC experts received thorough training and revision moments were provided to discuss certain issues in group.

The sampling method differed between the two countries, both having their limitations. For Belgium, only a subsample of the DCCs were recruited and for the Netherlands a non-probability sampling was used with similar coverage of the six regions involved. The representativeness of the DCCs operating in these two regions can be questioned, although we do not expect a strong association between the selection of DCCs and prevalence of AMR. Moreover, only in a part of both countries, DCCs were recruited to participate in the study for practical reasons (Flanders represents 58% of inhabitants of Belgium and the southern provinces in the Netherlands 23%), which may compromise the generalizability of the results.

Detailed information about the use of antimicrobials and hospital admissions is lacking, which might have been of added value to further explore the difference in antimicrobial use and hospital admissions between both countries.

## Conclusion

The prevalence of ESBL-E, the prevalence of CipR-E, the use of antimicrobial agents and hospital admissions among children attending a DCC was significantly higher in Belgium, compared to the Netherlands. Children who travelled to Asia within the previous 6 months had a higher odds to be a carrier of ESBL-E and children who received an antimicrobial treatment within the previous 6 months, had a higher odds to be a carrier of CipR-E. Cleaning the changing mat after each use was a protective factor for CipR-E carriage. The differences between the two countries should be further studied in order to improve the policy on antimicrobial use and hospital admissions in children. Setting up a surveillance study in the community might give additional information about carriage of antimicrobial resistance in the community and give some insight into possible risk factors.

### Supplementary Information

**Additional file 1.**

## Data Availability

The datasets used and/or analysed during the current study are available from the corresponding author on reasonable request.

## Abbreviations

AMR: Antimicrobial resistance
ATP: Adenosine triphosphate
CiPR-E: Ciprofloxacin-resistant *Enterobacterales*
CPE: Carbapenemase-producing *Enterobacterales*
DCC: Day care centre
ESBL-E: Extended-spectrum beta-lactamase-producing *Enterobacterales*
ICC: Intraclass correlation coefficient
IPC: Infection prevention and control
IRIS: Infection RIsk Scan
OR: Odds ratio
RLU: Relative light units
RR: Risk ratio
SE: Standard error
STROBE: STrengthening the Reporting of OBservational studies in Epidemiology
VRE: Vancomycin-resistant enterococci
95% CI: 95% Confidence interval
