# Supplementary material for: Cross-border differences in the prevalence and risk factors for carriage of antimicrobial resistance in children attending daycare centers: a point prevalence study in the Netherlands and Belgium
Source: BMC Infect Dis. 2024 Jan 24;24:131. doi: 10.1186/s12879-024-08996-9 (PMC10809597; doi:10.1186/s12879-024-08996-9)
Supplement: Supplementary file 1 — Additional file 1. [file 12879_2024_8996_MOESM1_ESM.docx]

**Supplementary material to support “Prevalence and Risk Factors for Carriage of antimicrobial resistance in Children Attending Daycare Centres: A Point Prevalence Study in the Netherlands and Belgium”**

**Additional file 1: Risk factors for carriage of extended-spectrum beta-lactamase-producing *Enterobacteriaceae* and ciprofloxacin-resistant *Enterobacteriaceae*: univariate logistic regression**

|  | Extended-spectrum beta-lactamase-producing *Enterobacteriaceae* | | | Ciprofloxacin-resistant *Enterobacteriaceae* | | |
| --- | --- | --- | --- | --- | --- | --- |
| Reference category is ‘yes’ | OR | 95% CI (Wald) | p-value | OR | 95% CI (Wald) | p-value |
| Age | 0.88 | (0.69 – 1.11) | 0.28 | 1.14 | (0.91 – 1.42) | 0.26 |
| Days per week attending the DCC | **1.52**** | **(1.30 – 1.80)** | **<0.001** | **1.26** | **(1.08 – 1.46)** | **<0.01** |
| Gender | 1.25 | (0.82 – 1.91) | 0.29 | 0.15 | (0.77 – 1.70) | 0.50 |
| Antimicrobial use (<6 months) | **0.53** | **(0.35 – 0.80)** | **0.002** | **0.63** | **(0.42 – 0.93)** | **0.02** |
| Hospital admission (<6 months) | 1.11 | (0.57 – 2.10) | 0.76 | 0.74 | (0.42 – 1.29) | 0.28 |
| Parental occupation; at least one parent is working in care | 1.26 | (0.78 – 2.03) | 0.35 | 1.10 | (0.65 – 1.55) | 0.98 |
| Animal contact (<6 months) | **2.10** | **(1.30 – 3.30)** | **0.002** | **1.62** | **(1.04 – 2.52)** | **0.03** |
| Animal contact (<6 months) – at home | **1.85** | **(1.20 – 2.80)** | **0.005** | 1.41 | (0.93 – 2.13) | 0.10 |
| Animal contact (<6 months) – zoo | 1.54 | (0.97 – 2.47) | 0.07 | 1.36 | (0.89 – 2.09) | 0.16 |
| Animal contact (<6 months) – live stock | 2.77 | (0.85 – 9.02) | 0.09 | 1.37 | (0.61 – 3.08) | 0.49 |
| Travel abroad (<6 months) | **0.62** | **(0.4 – 0.95)** | **0.03** | 1.00 | (0.68 – 1.49) | 0.99 |
| Travel abroad (<6 months) Africa | **0.27** | **(0.09 – 0.79)** | **0.02** | 1.04 | (0.23 – 4.60) | 0.96 |
| Travel abroad (<6 months) – Asia | **0.07** | **(0.02 – 0.27)** | **<0.001** | **0.20** | **(0.06 – 0.73)** | **0.01** |
| Travel abroad (<6 months) – Europe | 0.75 | (0.49 – 1.14) | 0.18 | 1.06 | (0.72 – 1.57) | 0.76 |
| Travel abroad (<6 months) – Latin-Amerika | >999.9 | (<0.001 - >999.9) | 0.98 | 1.25 | (0.16 – 9.94) | 0.83 |
| Travel abroad (<6 months) – North-Amerika | >999.9 | (<0.001 - >999.9) | 0.99 | >999.9 | (<0.001 - >999.9) | 0.99 |
| Country (Belgium) | **0.34** | **(0.22 – 0.53)** | **<0.001** | **0.39** | **(0.26 – 0.60)** | **<0.001** |
| Environmental cultivation |  |  |  |  |  |  |
| *ATP toys* | 1.14 | (0.85 – 1.52) | 0.38 | 0.99 | (0.76 – 1.30) | 0.94 |
| *ATP sanitary* | 1.23 | (0.87 – 1.73) | 0.24 | 0.84 | (0.60 – 1.17) | 0.30 |
| *ATP group* | 1.30 | (0.94 – 1.82) | 0.12 | 0.96 | (0.71 – 1.32) | 0.81 |
| *ATP kitchen* | 1.01 | (0.79 – 1.46) | 0.64 | 0.95 | (0.70 – 1.29) | 0.75 |
| Toilet Hygiene |  |  |  |  |  |  |
| *Toilet at child height* | 2.14 | (0.70 – 6.53) | 0.18 | 1.64 | (0.54 – 4.98) | 0.38 |
| *Sink at child height* | **2.94** | **(1.65 – 5.22)** | **<0.01** | 1.46 | (0.80 – 2.66) | 0.22 |
| *Liquid soap available at sink at child height* | **2.63** | **(1.49 – 4.63)** | **<0.01** | **2.09** | **(1.26 – 3.47)** | **<0.01** |
| *Changing mat cover is intact and easy to clean* | 1.08 | (0.38 – 3.14) | 0.88 | 0.42 | (0.10 – 1.79) | 0.24 |
| *Changing mat is cleaned after each use* | 1.59 | (0.98 – 2.60) | 0.06 | **1.58** | **(1.01 – 2.45)** | **0.04** |
| *Used diapers are immediately put in the diaper container* | 0.17 | (0.02 – 1.24) | 0.08 | 0.59 | (0.21 – 1.68) | 0.32 |
| *Liquid soap available at sink next to changing mat* | 1.06 | (0.31 – 3.59) | 0.92 | 1.27 | (0.43 – 3.74) | 0.67 |
| *Liquid soap available at sink staff toilet* | **2.5** | **(1.31 – 4.82)** | **<0.01** | 0.79 | (0.33 – 1.87) | 0.58 |
| *No potties are used* | 0.89 | (0.52 – 1.51) | 0.66 | 1.41 | (0.85 – 2.34) | 0.18 |
| *Disposable paper towels are available at all sinks* | 1.05 | (0.64 – 1.71) | 0.860 | 1.08 | (0.68 – 1.72) | 0.74 |
| Food Hygiene |  |  |  |  |  |  |
| *Maximum temperature of fridge >= the national guideline* | **1.91** | **(1.18 – 3.10)** | **<0.01** | **1.61** | **(1.03 – 2.53)** | **0.04** |
| *Child-bonded bottles and teats* | 1.18 | (0.71 – 1.97) | 0.52 | 0.74 | (0.22 – 2.48) | 0.63 |
| *After use, bottles are immediately rinsed* | 0.87 | (0.52 – 1.45) | 0.59 | 0.32 | (0.83 – 2.11) | 0.24 |
| *Only formula in powdered form is accepted* | 1.79 | (0.39 – 8.34) | 0.46 | <0.001 | (<0.001 - >999.9) | 0.98 |
| *Food preparation separated from changing area* | 1.53 | (0.34 – 7.02) | 0.58 | 1.31 | (0.29 – 5.98) | 0.73 |
| *Breast milk defrosted in the fridge* | **1.90** | **(1.00 – 3.60)** | **0.05** | 0.67 | (0.30 – 1.51) | 0.33 |
| *No foods passed expiry date* | 1.14 | (0.61 – 2.13) | 0.67 | 1.55 | (0.91 – 2.66) | 0.11 |
| *Cleaning schedule for the kitchen* | 0.59 | (0.34 – 1.03) | 0.06 | 1.40 | (0.91 – 2.17) | 0.13 |
| *Dish cloths, towels and tea towels are not visually soiled* | **0.31** | **(0.11 – 0.86)** | **0.02** | 0.58 | (0.27 – 1.22) | 0.15 |
| Hand hygiene and preconditions |  |  |  |  |  |  |
| *Hand hygiene education for children* | 1.07 | (0.62 – 1.86) | 0.81 | 1.26 | (0.76 – 2.10) | 0.37 |
| *Children wash their hands after going to the toilet* | 1.73 | (0.98 – 3.04) | 0.06 | **2.29** | **(1.35 – 3.86)** | **<0.01** |
| *Children wash their hands after playing outside* | 1.18 | (0.69 – 2.04) | 0.54 | 0.86 | (0.51 – 1.44) | 0.56 |
| *Children wash their hands before eating* | 1.4 | (0.82 – 2.38) | 0.21 | 1.23 | (0.75 – 2.00) | 0.41 |
| Staff wash their hands after changing a diaper of cleaning the nose/bum of a child | 1.37 | (0.90 – 2.11) | 0.15 | 1.39 | (0.94 – 2.06) | 0.10 |
| Staff wash their hands after going to the toilet | **11.64** | **(5.67 – 23.88)** | **<0.001** | 1.30 | (0.49 – 3.47) | 0.60 |
| Staff does not wear rings | **0.59** | **(0.39 – 0.90)** | **0.01** | **0.47** | **(0.31 – 0.69)** | **<0.001** |
| Staff does not wear wrist jewellery | 1.13 | (0.74 – 1.73) | 0.56 | 0.74 | (0.50 – 1.09) | 0.12 |
